# Assessment of oxidative stress in saliva of children with dental erosion

**DOI:** 10.1590/S1679-45082018AO4203

**Published:** 2018-06-05

**Authors:** Caleb Shitsuka, Flávia Kazue Ibuki, Fernando Neves Nogueira, Fausto Medeiros Mendes, Marcelo Bönecker

**Affiliations:** 1 Faculdade de Odontologia, Universidade de São Paulo, São Paulo, SP, Brazil.

**Keywords:** Tooth erosion, Saliva, Oxidative stress, Pediatric dentistry, Erosão dentária, Saliva, Estresse oxidativo, Odontopediatria

## Abstract

**Objective:**

To evaluate oxidative stress in saliva of children with dental erosion as compared to children with no erosion.

**Methods:**

One single examiner, trained and prepared to make diagnosis of dental erosion according to the Basic Erosive Wear Examination index, selected 40 children aged 4 to 6 years, who attended a pediatric dentistry prevention clinic. Two groups were formed - one comprising children with dental erosion (n=22), and another with no dental erosion (n=18). The quantity of dental biofilm was verified using the Simplified Index of Oral Hygiene, and unstimulated saliva was collected for biochemical analyses. The following were assessed in saliva: flow rate, buffering capacity, pH, and total protein concentration. Malondialdehyde levels were also verified to determine oxidative stress and total antioxidant status.

**Results:**

The quantity of biofilm was smaller in children with mean dental erosion±standard deviation (0.76±0.25), as compared to those with no dental erosion (1.18±0.28). There was no statistical difference in saliva parameters of oxidative stress in children with dental erosion.

**Conclusion:**

The activity of oxidative stress in saliva did not influence dental erosion process when in its early stages.

## RESUMO

**Objetivo:**

Avaliar o estresse oxidativo da saliva de crianças que possuíam erosão dentária, comparadas àquelas que não apresentavam esta situação.

**Métodos:**

Um único examinador, treinado e calibrado para o diagnóstico de erosão dentária, segundo o índice de *Basic Erosive Wear Examination*, selecionou 40 crianças de 4 a 6 anos de idade que frequentavam uma clínica de prevenção de odontopediatria. Dois grupos foram formados - um com aquelas que apresentavam erosão (n=22) e outro sem erosão (n=18). A quantidade do biofilme dental foi obtida utilizando o Índice de Higiene Oral Simplificado, tendo sido feita a coleta de saliva não estimulada para as análises bioquímicas. O fluxo salivar, a capacidade tampão da saliva, o pH salivar e a proteína total da saliva foram avaliados. Também foi verificado o valor do malondialdeído para determinação do estresse oxidativo e o total antioxidante.

**Resultados:**

A quantidade de biofilme foi menor nas crianças, com erosão dentária média±desvio padrão (0,76±0,25) comparadas àquelas sem erosão dentária (1,18±0,28). Não houve diferença estatística nos parâmetros salivares de estresse oxidativo em crianças com erosão dentária.

**Conclusão:**

A ação do estresse oxidativo na saliva não influenciou na erosão dentária, quando ainda nos estágios iniciais.

## INTRODUCTION

Dental erosion is an oral health problem that affects mainly children and adolescents due to recent changes in lifestyle.^(^ ^1^ ^)^ It is caused by a chemical process of irreversible loss of the mineral and superficial structure of teeth, resulting from an acid aggression with no bacterial involvement.^(^ ^2^ ^)^ Its etiology is complex and multifactoral; it may be of intrinsic origin, when associated with the presence of gastric acid in the oral cavity, or of extrinsic origin, caused mainly by the high consumption of acid foods and beverages.^(^ ^3^ ^)^ In the last few years, dental erosion has been in the spotlight, mainly due to its high and increasing prevalence. This problem has become a concern for patients, especially when it reaches a more advanced stage, causing functional and cosmetic losses, as well as discomfort. In these cases, treatment becomes a challenge for health professionals.^(^ ^3^ ^)^

To avoid worsening of this problem, dental surgeons can use preventive measures, such as therapeutic use of fluorides and dietary education. The body itself has an important form of natural protection present in the oral environment, which is salivary fluid.^(^ ^4^ ^)^

Saliva acts in several ways in the process of protecting the teeth against tooth erosion, since it has many physical and chemical properties, performing specific functions to protect the dental structure, such as dilution of erosive acid substances in the oral cavity through salivary flow, neutralizing and buffering of acids by pH, and supply of calcium and phosphate ions.^(^ ^5^ ^,^ ^6^ ^)^

An important protective function present in saliva and still little studied is the antioxidant system responsible for controlling oxidative damage. It is considered the first line of defense for oxidative stress, which can cause cell damage and lead to cell death.^(^ ^7^ ^)^

When oxidative stress is present in the oral environment, cell damage is greater, causing alteration in the formation and possible decrease of dental biofilm, which is considered a protective factor against dental erosion, acting as a mechanical barrier. The decreased amount of dental biofilm reduces protection against acid aggression in the teeth, and may become an aggravating factor for this oral health problem.^(^ ^7^ ^)^

This oxidative stress has been identified as an important contributor to several inflammatory, chronic and degenerative diseases, including oral diseases, such as caries and periodontal diseases. Nevertheless, little is known about the activity of the antioxidant system in children with dental erosion.

## OBJECTIVE

To evaluate oxidative stress in saliva of children with dental erosion as compared to those who do not present with oral health problems.

## METHODS

This cross-sectional study was approved by the Internal Review Board of the *Faculdade de Odontologia da Universidade de São Paulo* (USP), under protocol number 177.451, CAAE: 11100412.7.0000.0075.

The study sample comprised 40 children aged 4 to 6 years, who attended the Pediatric Dentistry Prevention Clinic of the *Faculdade de Odontologia* - USP, in 2013 and 2014. The sample was divided into two groups - the first (n=18) had children with no dental erosion, and the second group (n=22), children with erosion.

According to the criteria established, children and their respective legal representatives who attended the clinic were invited to participate. The representatives who accepted the invitation filled in and signed the Informed Consent Form, and the children were examined.

The amount of dental biofilm was measured using the Simplified Oral Hygiene Index (OHI-S), which evaluates the surface of some teeth in order to measure the amount of biofilm and the patient’s oral hygiene.^(^ ^8^ ^)^ The clinical examination was conducted using a plaque disclosing agent (fuchsin 3%), applied to the dental surfaces with a cotton swab.

The diagnosis of dental erosion was performed by a single trained examiner. The examiner underwent two four-hour sessions of training and calibration with exercises for diagnosing erosion, using 20 images of clinical photographs and 20 teeth extracted and provided by the Bank of Human Teeth of the *Faculdade de Odontologia* - USP, with different levels of wear. The Kappa test for intra-examiner reliability was 0.89.

Relative isolation was used in the clinical examination to diagnose erosion, with cotton balls and air-jet drying, dental mirror #5 and lightning by reflector. The Basic Erosive Wear Examination (BEWE) index was used to record the erosion lesions found.^(^ ^9^ ^)^

After the clinical examination, children were invited to come back another day in order to collect saliva in an standardized way, so that there would be no alterations in relation to content and circadian rhythm. All children were previously informed to not do oral hygiene and to fast for at least 2 hours before saliva collection.

The non-stimulated salivary sample was collected always between 2pm and 4pm. Prior to collection, each child was given a glass of distilled water to make a 30-second mouthwash and spit. Later, the child was oriented to accumulate saliva on the floor of the mouth and let it drip into a graduated plastic tube for 10 minutes, controlled by digital timer.

The evaluation of saliva flow, buffer capacity and pH was made shortly after collecting saliva and recorded in a standardized form. Immediately after collection, the samples were kept on ice and then stored under low temperatures (-80^°^C) to keep their chemical properties.

During collection, the saliva flow rate was determined by the ratio collected volume and time (10 minutes), expressed as mL/minute.

Next, a graduated glass pipette was used to collect 1mL of the saliva kept in the graduated plastic tube. This sample of 1mL of saliva was placed in another graduated plastic tube, which had also been kept on ice at low temperatures.

The UltraBASIC UB-10 digital portable pH meter (Denver Instrument, Bohemia, NY), was used to estimate the baseline salivary pH, after being calibrated by dipping the electrode in two different solutions with known pH values, according to the manufacturer’s instructions.

The buffer capacity was determined by titration with a 0.01N HCl solution, immediately after collecting the saliva sample and assessing flow rate and baseline pH. Using a glass pipette, 0.2mL of 0.01N HCl were added to the 1mL sample of saliva separated in the graduated plastic tube. The tip of the pH meter (electrode) was washed with distilled water and dried with absorbent paper, so that a new measure would be performed, now with the acid added to the salivary sample. This process was always repeated with the addition of 0.2mL of HCl, and the pH value was recorded in the clinical form until achieving a pH value ≤5.5.

Data on the total buffer capacity were obtained, and the three main buffers with pH scale values were represented: up to 7, between 6.9 and 6, and between 5.9 and 5.5. The remaining amount of saliva samples was stored at -80^°^C for further evaluation in the laboratory.

The total antioxidant status (TAS) was determined according to the instructions contained in the TAS Kit (Randox, UK). This method consists of incubating the ABTS reagent with peroxidase and hydrogen peroxide to produce the ABTS radical, whose stable color is bluish-green and can be measured at 600nm. In order to apply the method, plastic cuvettes were used, and the reaction was monitored in duplicates by spectrophotometry.

Oxidative stress was determined by lipid peroxidation and followed the method described by Karatas et al.,^(^ ^10^ ^)^ which is based on the determination of malondialdehyde (MDA) using high-performance liquid chromatography (HPLC).

Sample preparation was done by homogenizing 0.5mL of saliva sample in 1.75mL of 0.4M perchloric acid, for 1 minute. This mixture was kept on ice for 30 minutes, and subsequently centrifuged at 27,000g, for 10 minutes. The supernatant fluid was removed and neutralized with 5M K_2_CO_3_ (4uL for 50uL of liquid), then put on hold for 1 hour, at -80^°^C.

In order to prepare the patterns, 5uL of tetraethoxypropane were diluted in 5mL of 0.1N HCl. From this solution 0.5mL were removed in 50mL of H_2_O.

The reading was done in duplicate using HPLC, with reading of 254nm, with total time of 5 minutes.

### Statistical analysis

All data (clinical and laboratory data sheets) were input in a Microsoft Excel spreadsheet and later verified by the researcher. For statistical analysis, the data were transferred to the software Stata version 9.0 (Stata Corp LP, College Station, USA). Initially, the Kolmogorov-Smirnov test of normality was conducted to compare the groups of children with dental erosion and the group of children with no dental erosion, followed by the Student’s *t* test. For all variables studied, the significance level was set at 5%.

## RESULTS

A total of 40 children participated in the study – in that, 22 (55%) were female, 22 (55%) had dental erosion, and 18 (45%) had no dental erosion. For the group of children with dental erosion the mean age±standard deviation was 5.50±0.74 years, and for the group with no erosion, 5.16±0.85 years.

The mean OHI-S (amount of biofilm) calculated by the Student’s *t* test was lower (p<0.0001) for children with dental erosion, as compared to those with no erosion (Table 1).

**Table 1:** Analysis of factors possibly related to dental erosion in children

Variables	No erosion Mean (SD)	With erosion Mean (SD)	p value*
OHI-S	1.18 (0.28)	0.76 (0.25)	<0.0001
Salivary flow, mL/minute	0.26 (0.09)	0.26 (0.07)	0.998
Baseline pH	7.16 (0.14)	7.26 (0.23)	0.107
Total buffering capacity, mL (HCl 0.01N)	1.12 (0.21)	1.23 (0.17)	0.071
Buffering capacity according to pH range, mL (HCl 0.01N)			
pHb -7.0	0.30 (0.14)	0.39 (0.20)	0.124
6.9-6.0	0.55 (0.16)	0.62 (0.18)	0.209
5.9-5.0	0.26 (0.09)	0.22 (0.07)	0.145

* Calculated using the Students’s *t* test (p<0.05). SD: standard deviation; OHI-S: Simplified Oral Hygiene Index.

The variables related to the antioxidant system are depicted in table 2. There were no alterations for MDA and TAS values.

**Table 2:** Analysis of the total antioxidant status and oxidative stress related to dental erosion in children

Variables	No erosion	With erosion	p value*
	Mean (SD)	Mean (SD)	
MDA, nM/mL	1,427.5 (1,701.0)	2,895.4 (4.057,6)	0.178
TAS, nM/mL	5,559.2 (5,839.2)	15,332.06 (35,372.4)	0.255


* Calculated using the Student’s *t* test (p<0.05). SD: standard deviation; MDA: malondialdehyde; TAS: total antioxidant status.

## DISCUSSION

Despite the high prevalence of dental erosion in the population, especially in children and young people, this oral health problem is mostly found in early stages, in which the lesions are usually limited to the enamel.^(^ ^11^ ^-^ ^13^ ^)^ In our study, of the 22 children diagnosed with erosion by the BEWE index, 86% had low risk of dental erosion.

Seen as the natural protection of the dental surface, dental biofilm can act as a physical barrier against the erosive acid attack,^(^ ^7^ ^,^ ^11^ ^,^ ^14^ ^)^ and act selectively, avoiding calcium and phosphate loss.^(^ ^15^ ^)^ This understanding is in tune with the findings of our study, in which children with dental erosion had a smaller amount of biofilm (p<0.0001), with mean±standard deviation being 0.76±0.25, as compared with the children without erosion (1.18±0.28).

Other changes may occur in the oral cavity, such as in pH and temperature, which influence the oral microbiota and may modify the dental biofilm.^(^ ^16^ ^)^ Regarding the salivary pH, there was no difference between the groups studied, in accordance with previous studies.^(^ ^17^ ^-^ ^20^ ^)^

The mean salivary flow rate for unstimulated collection of saliva in children with and without dental erosion was 0.26mL/minute, which corresponds to the mean of the general population (0.3mL/minute),^(^ ^21^ ^)^ taking into account that salivary flow rate in deciduous dentition is lower than in permanent dentition.^(^ ^22^ ^)^ These findings corroborate other studies that demonstrated no correlation between salivary flow and dental erosion.^(^ ^17^ ^,^ ^18^ ^,^ ^20^ ^)^ Only one study demonstrated that the mean salivary flow of those with dental erosion was lower than of those who did not have this health problem.^(^ ^19^ ^)^

The buffering capacity of saliva has the protective function of pH resistance to acid induction. Our data did not show a correlation between this protective capacity and children with dental erosion, corroborating other studies.^(^ ^17^ ^-^ ^19^ ^)^

The sample of our study comprised children aged 4 to 6 years, with deciduous dentition. This choice was made in order to minimize the alterations of the antioxidant system in face of inflammation that could exist in the process of tooth eruption.^(^ ^23^ ^)^

The analyses of TAS and oxidative stress showed no statistical difference in the unstimulated saliva of children with dental erosion. Most children were diagnosed as having a low risk of erosion, therefore the role played by TAS and oxidative stress are not known in the presence of more severe injuries.

It was not possible to correlate TAS and oxidative stress in the saliva of children with a severe risk of dental erosion due to a limitation of the study, which adopted a convenience sample. Possibly, in more advanced stages of tooth erosion, the most superficial layer formed from a demineralized organic matrix on the dentin, which acts as a protective layer against the progression of mineral loss,^(^ ^24^ ^-^ ^25^ ^)^ could suffer influence of oxidative stress and TAS present in saliva.

Although the direct action of TAS and oxidative stress did not influence dental erosion, they may have influenced the formation of dental biofilm. This is pointed out in some studies in which the antioxidant system acts as a regulator in the formation of the dental biofilm and, therefore, is related to some oral diseases, such as periodontal diseases and decay.^(^ ^26^ ^-^ ^28^ ^)^ Further studies are necessary to understand this possible correlation in patients with erosion.

## CONCLUSION

Children with dental erosion had less dental biofilm than those with no erosion. There was no difference in the total antioxidant status or in malondialdehyde levels (oxidative stress) in saliva of children with early stages of dental erosion.
